# Purification, biochemical characterization and self-assembled structure of a fengycin-like antifungal peptide from *Bacillus thuringiensis* strain SM1

**DOI:** 10.3389/fmicb.2013.00332

**Published:** 2013-11-21

**Authors:** Anupam Roy, Denial Mahata, Debarati Paul, Suresh Korpole, Octavio L. Franco, Santi M. Mandal

**Affiliations:** ^1^Central Research Facility, Indian Institute of Technology KharagpurKharagpur, India; ^2^Amity Institute of Biotechnology, Amity University, Sector 125Noida India; ^3^Council of Scientific and Industrial Research, Institute of Microbial Technology, Sector 39AChandigarh, India; ^4^Centro de Análises Proteômicas e Bioquímicas, Pós-Graduação em Ciências Genômicas e Biotecnologia UCBBrasília, Brazil

**Keywords:** antimicrobial activity, *Bacillus thuringiensis*, fengycin, self-assembled structure

## Abstract

An antifungal lipopeptide fengycin, producing strain SM1 was isolated from farm land soil sample and identified as *Bacillus thuringiensis* strain SM1 by using 16S rDNA analysis. Fengycin detected in the culture extract was further purified using HPLC and showed a molecular mass of 1492.8 Da by MALDI-TOF-MS analysis. Purified fengycin was allowed to construct their self-assembled structure onto a hydrophobic surface showing a clear improvement of antibacterial activity. In self-assembly, fengycin adapts a spherical micelle core shell like structure. Self-assembled fengycin may be a successful antimicrobial compound modifying its action from confined antifungal function. Besides it can open up a new area of research in supramolecular lipopeptide based compound making. This can revealed the mode of action of this unique self-assembled structure to fully evaluate its potential for use as an antimicrobial drug to control the emergence of bacterial infection.

## INTRODUCTION

The increasing tendency of microbial infections, rapid emergence of drug-resistant to recent antibiotics and quick evolution through mutation are of great threats to control of microbial infection (Samanta et al., 2013). Infectious disease has become the biggest killer among children and young adults, and has now become a burden to global economics and public health (Jones et al., 2008). This situation is becoming uncontrollable due to unique gained properties of genetically altered pathogens assigned with their unusual clinical symptoms (Gilsdorf and Zilinskas, 2005). Impediment in usual diagnostic and clinical evaluations or preventive strategies forces the modern research to overcome this fatal situation. Research strategies like applying of naturally occurring antimicrobial peptides (AMPs), combined administration of antibiotic agents (Anantharaman et al., 2010), and structural modification of antibiotics are extensively studied (Roy et al., 2013). Furthermore, structural modification of AMPs by self-assembly mechanism is reported to enhance the spectrum of the AMPs (Fernandez-Lopez et al., 2001).

Fengycin, a cyclic lipodecapeptide produced by *Bacillus subtilis* strain, containing a β-hydroxy fatty acid with a side-chain length of 16–19 carbon atoms offers an efficient antifungal activity. Like most the natural AMPs, fengycin seems to acts by improving the plasma membrane permeability of the target cell (Vanittanakom et al., 1986). This AMP is known to exhibit strong fungitoxic activity specifically against filamentous fungi, inhibiting enzymes phospholipase A2 and aromatase (Loeﬄer et al., 1986; Steller and Vater, 2000). Otherwise this peptide also cause hemolytic activity 40-fold lower than that of surfactin, showing a clear disadvantage (Hbid, 1996; Schneider et al., 1999; Hathout et al., 2000). In this study, an antifungal peptide fengycin was purified from an *B. thuringiensis* strain SM1 soil isolate. This lipopeptide has been characterized and checked for its antifungal activity against *Candida albicans*. Additionally, self-aggregation and arrangement of the fengycin molecule were also studied to check whether interfacial modification can enhance the spectrum of the microbial action or not.

## MATERIAL AND METHODS

### IDENTIFICATION OF THE STRAIN

One gram of farm land soil sample, collected from agricultural farm of IIT-Kharagpur campus, India, was suspended in 9 ml of sterile distilled water. Then 100 μl of this was serially diluted and amplified on sterilized LB agar plates with the following composition (g/l): peptic digest of animal tissue, 5.0; beef extract, 1.5; yeast extract, 1.5; sodium chloride, 5.0; agar 15.0 (pH adjusted to 7.2). Colonies with inhibition zone in their surroundings were selected for study and streaked on to fresh nutrient agar (NA, HiMedia, India) medium plates. Upon testing their purity all isolates were preserved at -70°C in culture medium containing 50% (v/v) glycerol until further use. The tested strain SM1 was grown on tryptone soya agar (TSA) medium. Phenotypic properties including morphology, physiology and biochemical characteristics of the isolate were done using standard procedures (Baindara et al., 2013). The identity of strain SM1 was also confirmed by using 16S rRNA gene sequence (Korpole et al., 2006) blast search analysis. CLUSTAL-W program of MEGA version 5 was used to align the all 16S rRNA gene sequences of the nearest type strains (Tamura et al., 2011) downloaded from the NCBI database. Manual correction of alignment was done using BioEdit sequence alignment editor (Hall, 1999). Pair-wise evolutionary distances were calculated with the Kimura two-parameter (Kimura, 1980) and a neighbor-joining phylogenetic tree was constructed using the MEGA version 5.0. 1000 replicates were taken to find the stability of phylogenetic tree.

### EXTRACTION OF LIPOPEPTIDE

A combination of acid and solvent extraction procedure (Vater et al., 2002) was followed to isolate lipopeptide produced from the strain. Centrifugation (13,000 × *g*) for 15 min of the culture broth at 4°C resulted cells to pellet down. pH of the supernatant was adjusted to 2.0 by addition of concentrated HCl. Supernatant of pH 2 was allowed to stand at 4°C for 16 h and resulted in precipitation. The sample was then centrifuged (13,000 × *g*) for 20 min at 4°C. Precipitate was collected and further extracted with methanol by stirring for 2 h. The lipopeptide containing methanol was collected after filtration and vacuum-dried.

### PURIFICATION OF LIPOPEPTIDES

Purification of lipopeptides was carried out as mentioned in Mandal et al. (2013). Extracted lipopeptides was dissolved in methanol. Then we fractionate it by reverse phase- HPLC (Agilent 1100 series, CA, USA) with a ZORBAX 300-SB18 column (4.6 mm × 250 mm, particle size 5 μm), at a flow rate of 1 ml/min. The mobile phase components were (A) 0.1% TFA in water and (B) 0.1% TFA in 70% acetonitrile solution. The gradient of solvent B was used to run the column were : 0–60% for 0–45 min, 60–80% for 45–55 min and 80–100% for 55–60 min. Eluted peptide from the column were monitored at 215 nm in a diode array detector. Fractions according to peaks obtained during HPLC were collected using a fraction collector (GILSON, France) coupled with the system. Speed vacuum concentration was used to concentrate the sample and it was then tested for antimicrobial activity. The fractions or peaks that showed antibacterial activity were re-chromatographed in the same column under similar conditions, except solvent B was used as 100% acetonitrile with a gradient of 0–10% for 30 min. The peptide concentration was determined using the RP-HPLC conditions and calibrated with surfactin (Sigma-Aldrich, St. Louis,USA).

### MALDI-TOF-MS AND SEQUENCING

Molecular mass and MS/MS sequencing of the purified and active lipopeptides were performed using a Voyager time-of-flight mass spectrometer (Applied Biosystems, CA, USA). Peptides were incubated with 10% NaOH in methanol at room temperature for 16 h. Lactone ring present in lipopeptide in MS/MS sequencing was cleaved. Cleaved peptide was liophilizated and extracted with methanol, and allowed it for mass spectrometry analysis. Spectra were recorded in the post-source decay (PSD) ion mode as an average of 100 laser shots with a grid voltage of 75%. The reflector voltage was reduced in 25% steps and guide wire was reduced 0.02–0.01% with an extraction delay time of100 ns.

### FATTY ACID ANALYSIS BY GC-MS

Fatty acid content associated with the lipopeptides was assessed by incubating the peptides (5 mg of each) with 0.5 ml of 6 M HCl at 90°C for 18 h in sealed tubes for acid hydrolysis. Fatty acids were then extracted with ether and treated with 0.95 ml methanol and 0.05 ml of 98% H_2_SO_4_ at 65°C for 6 h. n-hexane extraction was then done to obtain fatty acid methyl esters. Then we analyzed it on GC-MS with a Clarus 500 GC (PerkinElmer, USA) using helium as carrier gas with a flow rate of 1.0 ml/min. The column temperature was then maintained at 120°C for 3 min and thereafter gradually increased (8°C/min) to 260°C.

### STRATEGY TO FORM SELF-ASSEMBLED FENGYCIN

Chloroform solution of fengycin molecule was prepared at a concentration of 0.1 mg ml^-1^ at neutral pH. The solution was then rotated in chloroform solution for 12 h. and further characterized.

### CIRCULAR DICHROISM AND FOURIER TRANSFORM INFRARED SPECTROSCOPY

Circular dichroism (CD) spectra were recorded using samples at 1% wt and pH 7 with a path length of 0.001 cm. Spectra were recorded at room temperature from 250 to 180 nm, with a 0.2 nm data pitch and a scan rate of 50 nm min^-1^ by Jasco-810 spectropolarimeter. Millidegrees of rotation were converted to molar residual ellipticity (MRE). For FTIR measurements, both the extract and the reduced colloidal solution were analyzed on a Perkin Elmer FTIR instrument in the diffused reflectance mode at a resolution of 4.0 cm^-1^.

### SCANNING ELECTRON MICROSCOPY

Aliquots (100 μL) of each gel were prepared and placed in a 24 well plate. Overnight chloroform rotated samples were then affixed to SEM pucks using conductive carbon tape. The pucks were sputter-coated with gold using a CRC-150 sputter coater and imaged using an FEI Quanta 400 ESEM at 20.00 kV.

### ANTIMICROBIAL ASSAYS AND MIC DETERMINATION

Minimum inhibitory concentration (MIC) values of fengycin for antifungal or antibacterial activities were determined as followed by Samanta et al. (2013). The bacterial strains *Staphylococcus epidermidis* NCIM 2493 and gram negative *Escherichia coli*, fungal strains *C. albicans* and *Aspergillus niger* were taken in the study. These strains were cultured according to their specifications. Both, fenzycin and self-assembled fengycin was used at a concentration range from 1 mg ml^-1^ to 1.95 μg ml^-1^to evaluate the antifungal and antimicrobial activity. Microtiter plate dilution assay (Baindara et al., 2013) was done to study the MIC. MIC values were determined where no visible growth was observed. All independent experiments were repeated four times.

## RESULTS AND DISCUSSION

### CHARACTERIZATION OF BACTERIAL STRAIN

**FIGURE 1:**
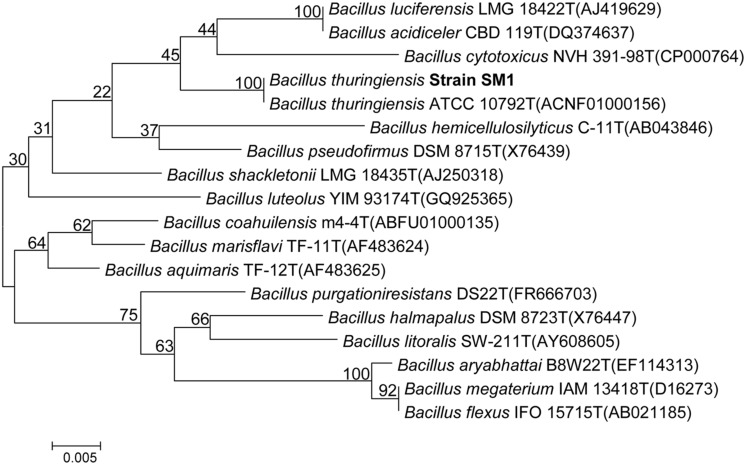
**Neighbor-joining phylogenetic tree based on 16S rRNA gene sequences, showing the phylogenetic relationship between *Bacillus thuringiensis* strain SM1 and other members of the genus *Bacillus*.** Bootstrap values (%) are given at the nodes.

Based upon colony morphology and upon zone of clearance we have selected a strain designated as SM1 in our study (data not shown). The prime focus of our study was to assess the molecular basis of its antimicrobial activity. In this course we have tried to identify the strain by phenotypic characteristics. Phenotypic and biochemical results revealed that the strain SM1 was a Gram-positive, rod shaped bacteria. It showed positive reaction for catalase activity and negative for oxidase activity and produced amylase. BLAST analysis of 16S rRNA gene sequence revealed significant identity (99.8%) with *B. subtilis* subsp. In aquosorum, a strain shown to produce fengycin-like lipopeptide. Neighbor joining phylogenetic tree which was constructed with 16S rRNA gene sequences of other members of the genus *Bacillus* gives confirmation of the strain *B. thuringiensis.* Result showed that a distinct cluster along with *B. thuringiensis* (**Figure 1**) is formed with a significant bootstrap value.

### PRODUCTION, PURIFICATION AND CHARACTERIZATION OF ANTIMICROBIAL PEPTIDE

**FIGURE 2:**
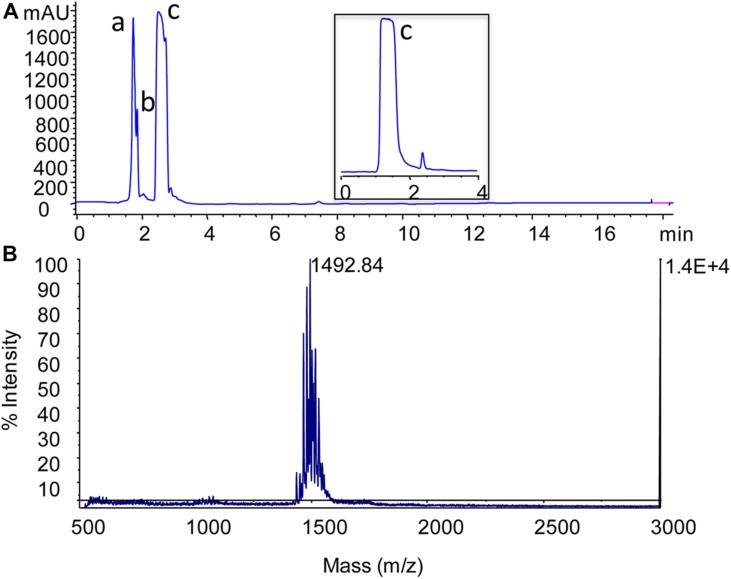
**Separation of the antimicrobial lipopeptide from acidic methanol extract by using reversed-phase HPLC.** Chromatogram profile of acidic methanol extract showed three peaks (a, b, and c) and among these fractions, fraction c (lipopeptide) showed antimicrobial activity **(A)**. MALDI-TOF mass spectrum of fengycin-like peptide **(B)**. Spectrum was acquired in positive ion linear mode and reproducibility of the spectrum checked several times with different spots of same sample.

Antimicrobial peptides were produced in conical flask for large scale preparation. The methanol extracts of lipopeptides obtained from the strain was screened for antifungal activity against*C. albicans*(data not shown) and subsequently purified using RP-HPLC. Methanol extract of sample showed multiple peaks during their HPLC analysis. Individual peaks fraction were collected and screened. Fraction 2 showed the highest antifungal activity. The fraction was further purified by a combination of chromatography techniques. The peptide obtained by affinity chromatography was purified by RP-HPLC (**Figure 2A**) and used to determine molecular mass by MALDI-TOF analysis. The peptide showed molecular mass of 1,492.84 (**Figure 2B**). The GC-MS analysis revealed the β-hydroxy fatty acid chain as C-18 long. Recently, Pathak et al. (2012) described details a series of fengycins from *Bacillus* species and also mentioned the production of new fengycin with molecular mass of 1492.8. The obtained fengycin (1492.84 Da) from SM1 strain is exactly identical with earlier reported fengycin from *B. subtilis* strain K1, as the amino acid composition of EOrnYTEVPEYV (Pathak et al., 2012).

### FORMATION OF SELF-ASSEMBLED STRUCTURE

Fengycin (0.1 mg ml^-1^ at neutral pH) was dissolved in chloroform and was rotated at 1000 rpm for 12 h to form their self-assembled structure using hydrophobic interaction. The circular dichroism spectroscopy, Fourier transform infrared spectroscopy, scanning electron microscopic techniques were used to characterize the self-assembled structure of fengycin. The antifungal and antibacterial activity of the self-assembled structure along with the purified one was assessed.

### CIRCULAR DICHROISM ANALYSIS

**FIGURE 3:**
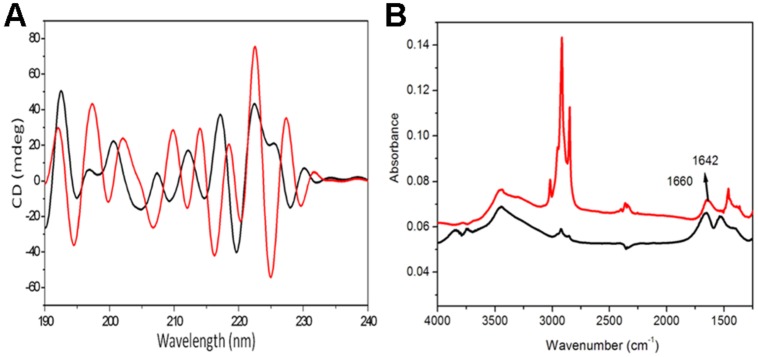
**Analytical confirmation of self-assembled structured of fengycin.** The deconvoluted CD spectra of the fengycin-like peptides rendering self-assembled cyclic conformation **(A)**; the black and red line represents pure fengycin-like peptide and their self assembled structure, respectively. FTIR spectra of fengycin **(B)**, for both cases the line graph indicates only pure fengycin (black line), fengycin after 12 h rotation (red line).

Circular dichroism is a sensitive method to the stereoisometry of amino acids constituting the peptide backbone. However, CD results of fengycin are useful, but they are not sufficient to draw a definitive conclusion about the conformation. As expected, this spectrum does not correspond to the conventional spectra of peptides which usually adopt α-helical or β-sheet conformation due to the cyclic structure of lipopeptide hindering that kind of conformation. This spectrum shows the broad positive band with peaks at the region of 218–227 nm and negative band centered at 205–211 nm (**Figure 3A**). It is expected that fengycin contains turns due to its closure ring structures of amino acids precluding the β-sheet or α-helical conformations. The presence of the positive band at 218 and 222 nm could be explained by the n-π* transition occurred within D-amino acids (Vass et al., 2001). Moreover, this band might also be due to an unconventional turn conformation adopted by the peptide cycle of fengycin (Vass et al., 1998, 2001, 2003, 2010). Another characteristic of the fengycin CD spectrum is the presence of negative bands at 206 and 211 nm, might be corresponding to the π-π* transition occurring within peptide bonds and is compatible with the presence of β sheet conformations. There are no significant positive band shifting at 222 nm in self-assembled fengycin which suggest the turn conformation present in self-assembled cyclic peptide. Another negative band at the region at 194–206 nm shows major shift in self assemble formation may be indicates the formation of “β sheet like micelles” in the structure (Vass et al., 2001).

### FTIR-ANALYSIS

Infrared spectrum of the revealed a broad stretching peak at 3441 cm^-1^, characteristic of peptides and at 3157.22 cm^-1^ reflects hydroxyl and amine groups (**Figure 3B**). Absorption around 2955 cm^-1^ is assigned to the symmetric stretch (–C–H) of CH_2_ and CH_3_ groups of aliphatic chains. Moreover, an absorption band at 1660 cm^-1^ depicted stretching mode of the CO–N bond and an intense stretching peak around 1470 cm^-1^ indicated the presence of ester carbonyl groups (C = O in COOH) in peptide (**Figure 3B**). The ester carbonyl group was proved from the band at 1266.23 cm^-1^ which corresponds to C–O deformation vibrations. Absorption around 1402 cm^-1^ was also characterized as aromatic group. The amide peak at 1660 cm^-1^ is shifted to 1642 cm^-1^ which suggest the aggregation of lipopeptide by the hydrogen bonding in between amide group.

**Table 1:** Antimicrobial activity of both pure and self-assembled fengycin.

Compounds	MIC (μg ml^-1^)			
	*C. Albicans.*	*A. niger*	*S. epidermidis*	*E. coli*
Self-assembled fengycin	7.81	15.62	125	125
Fengycin	15.62	15.62	1000	1000


### SEM IMAGE AND ANTIMICROBIAL ACTIVITY

**FIGURE 4:**
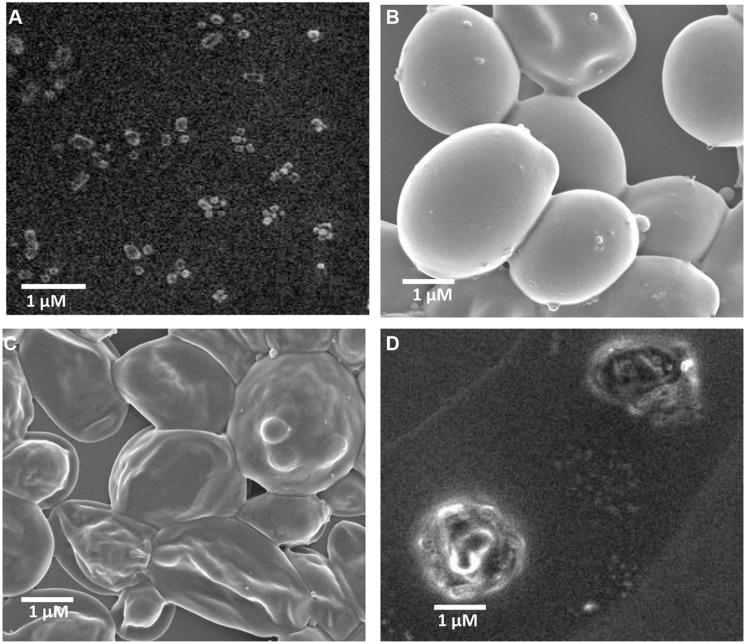
**Scanning electron micrograph of fengycin and activity against *C. albicans* cells.** Self -assembled globular micelle structure of fengycin **(A)**, scanning electron micrograph of *C. albicans* cells without treatment of fengycin peptide **(B)**, scanning electron micrograph of *C. albicans* cells after treatment of fengycin peptide **(C)**, scanning electron micrograph of *C. albicans* cells after treatment of self-assembled fengycin peptide **(D)**.

Self-assembled fengycin offered a globular micelle structure (**Figure 4A**). This self-assembled peptide showed same antifungal activity (MIC-15.62 μg ml^-1^) against *A. niger* compared with pure fengycin, whereas, activity was increased one fold when tested against *C. albicans* **Table 1**. Like most the natural AMPs, fengycin seems to act by making the plasma membrane of the target cell more permeable. Fengycin offers a concentration-dependent perturbing effect on the structural and morphological characteristics of DPPC monolayers (Deleu et al., 2005). Antifungal mechanism of fengycin appears to be driven mainly by the physicochemical properties of lipopeptide, i.e., its amphiphilic character and affinity for lipid bilayers (Deleu et al., 2008). To confirm the action on membranolytic or not, SEM images were taken from *C. albicans* cells with and without treatment of drug molecules. **Figure 4B** clearly shows that the surface of control group (without fengycin treatment) of fungus was smooth in texture whereas the morphology of the treated cells (**Figures 4C,D**) displayed significant perturbations, rough with number of blebs. Bleb formation following fengycin treatment suggested breakage in the contact between cell wall and membrane. It is quite interesting that self-assembled fengycin affected worst to the fungal strain (**Figure 4D**). This might be due to change in amphiphilic character and affinity of the self-assembled molecule. The amphiphilic character and affinity can change via self-assembled interaction (Hüttl et al., 2013). Beside one another interesting finding needs attention of further study. Generally fengycin molecule did not show any significant anti bacterial effect. But after self-assembly, it offered a minimum inhibition concentration of 500 μg ml^-1^ against both Gram-positive *S. epidermidis* and gram negative *E. coli*. This might be also due to shift of amphiphilic character and affinity resulting action against Gram-positive *S. epidermidis*.

In conclusion, many Gram-positive bacteria including *Bacillus* produce AMPs as defense molecule (Baindara et al., 2013). Here we are reporting a fengycin producing strain *B. thuringiensis*, isolated from soil sample. The strain showed highest similarity with *B. thuringiensis* strains related with fengycin isolation as *B. thuringiensis* CMB26 and BS8. The molecular mass of the lipopeptide is 1,493.84 suggested it is to be fengycin-like peptide. The isolated peptide was subjected to self-assembly. This strategy yields modified fengycin molecule which is active against bacteria. In summary self-assembled fengycin may be a successful antimicrobial compound modifying its action from confined antifungal function. Besides it can open up a new area of research in supramolecular lipopeptide based compound making. This can revealed the mode of action of this unique self-assembled structure to fully evaluate its potential for use as an antimicrobial drug to control the emergence of bacterial infection.

## Conflict of Interest Statement

The authors declare that the research was conducted in the absence of any commercial or financial relationships that could be construed as a potential conflict of interest.
